# Rapidly Developing Methicillin-Resistant Staphylococcus Aureus Pericarditis and Pericardial Tamponade

**DOI:** 10.7759/cureus.8001

**Published:** 2020-05-07

**Authors:** Laith Ali, Amre Ghazzal, Tariq Sallam, Brian Cuneo

**Affiliations:** 1 Internal Medicine, MedStar Washington Hospital Center, Washington, DC, USA; 2 Critical Care/Pulmonary Medicine, MedStar Washington Hospital Center, Washington, DC, USA

**Keywords:** pericardial diseases, hiv aids, methicillin resistant staphylococcus aureus (mrsa), pericardial tamponade

## Abstract

Methicillin-resistant staphylococcus aureus (MRSA) pericarditis is a rare life-threatening infection. A 46-year-old female with hypertension, acquired immunodeficiency syndrome (AIDS) and recurrent neck abscesses, presented with a neck abscess and sepsis. Bloody purulent drainage from the abscess was found and antibiotics were started. Drainage was positive for MRSA. Four days after, course was complicated by acute pericarditis and pericardial tamponade; pericardial fluid was drained and was positive for MRSA. Vancomycin was continued, and aspirin and colchicine were started. Two days later, there was a recurrent pericardial fluid collection with loculation. Surgery was thought to be dangerous in the setting of CD4 count of 12. She was managed conservatively thereafter, with vancomycin, aspirin and colchicine, and was successfully discharged from the hospital.

## Introduction

The pericardium is a fibroelastic sac composed of visceral and parietal layers separated by a potential space, the pericardial cavity, which can normally have up to 50 mL of fluid. Acute pericarditis is an inflammation of the pericardium. It has been reported in 0.1%-0.2% of hospitalized patients, and 5% of patients presenting to the emergency department with a nonischemic chest pain [1,2]. Pericardial disease can occur secondary to inflammatory, neoplastic, vascular, iatrogenic, and idiopathic causes [3-5]. Cardiac tamponade is one of the complications of acute pericarditis. In a case series, it occurred in 14% of patients with idiopathic pericarditis and 61% of patients with neoplastic, tuberculous, or purulent pericarditis [6].

Methicillin-resistant staphylococcus aureus (MRSA) pericarditis is a rare life-threatening infection, with only few cases reported in literature [7,8]. Mortality rates for purulent pericarditis were reported to be as high as 40% in treated population [9]. In immunosuppressed patients or following thoracic surgery, staphylococcus aureus (30%) and fungi (20%) are more common [10].

We report a case of a rapidly developing MRSA pericarditis and tamponade in a patient with acquired immune deficiency syndrome (AIDS).

## Case presentation

**Figure 1:**
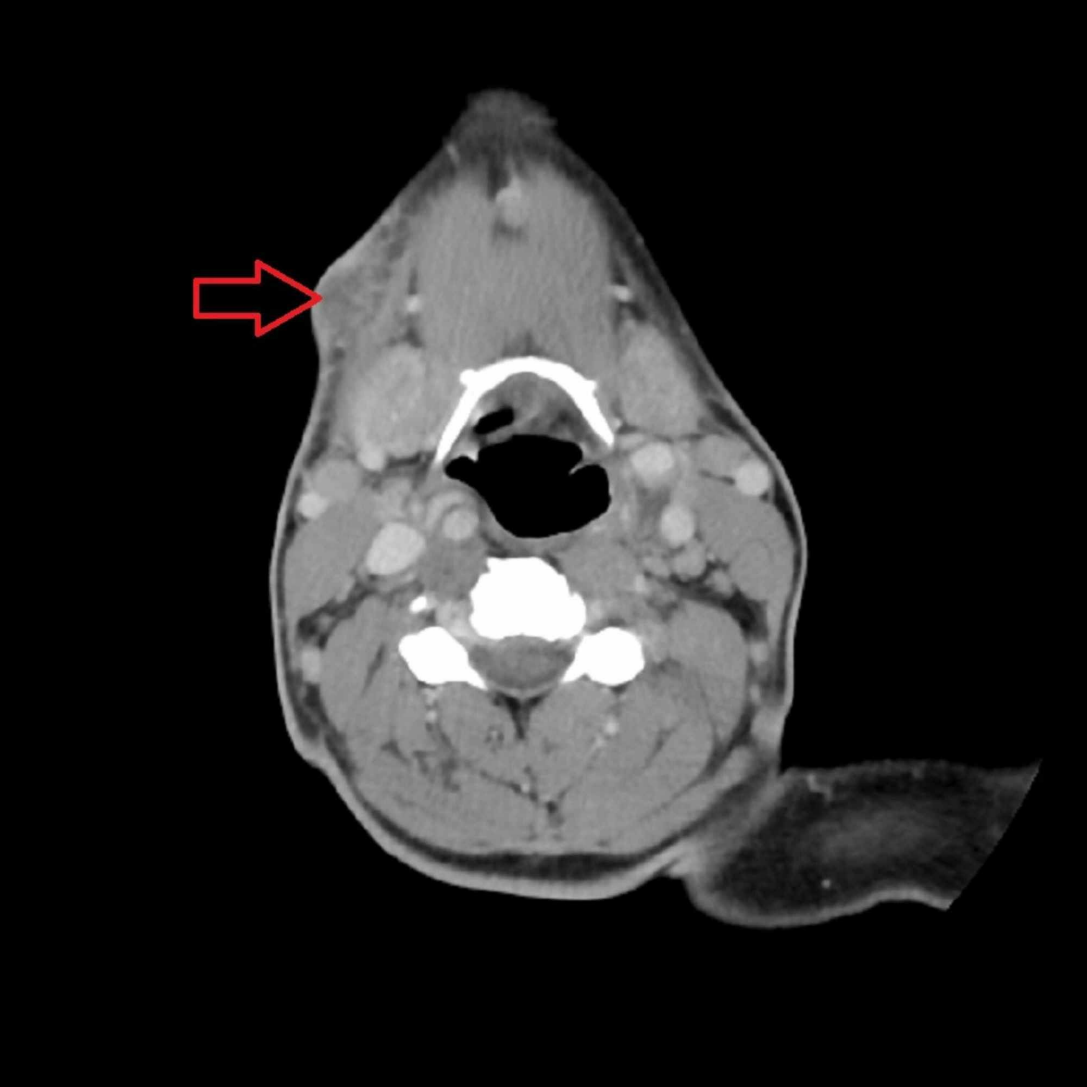
CT scan, showing the neck abscess in the anterior part on the right side.

A 46-year-old female with AIDS (CD4 count of 12), presented with fever and pleuritic positional chest pain. She was found to have a neck abscess and to be in sepsis (temperature 38.6 C, heart rate 114 bpm and white blood cell (WBC) count of 12.2 x 10^9^/L). Neck exam was remarkable for an anterior fluctuant neck mass. Cardiovascular exam on the day of admission showed regular rate and rhythm, normal heart sounds, and no jugular venous distention (JVD). CT scan of the neck was done, which showed the abscess on the right side (Figure 1). Her neck abscess was drained in the emergency department, and samples were sent for evaluation. Blood cultures were drawn, and the patient was started empirically on vancomycin, and piperacillin/tazobactam for treatment of sepsis. Blood and abscess fluid cultures grew MRSA. Thus, vancomycin was continued.

**Figure 2:**
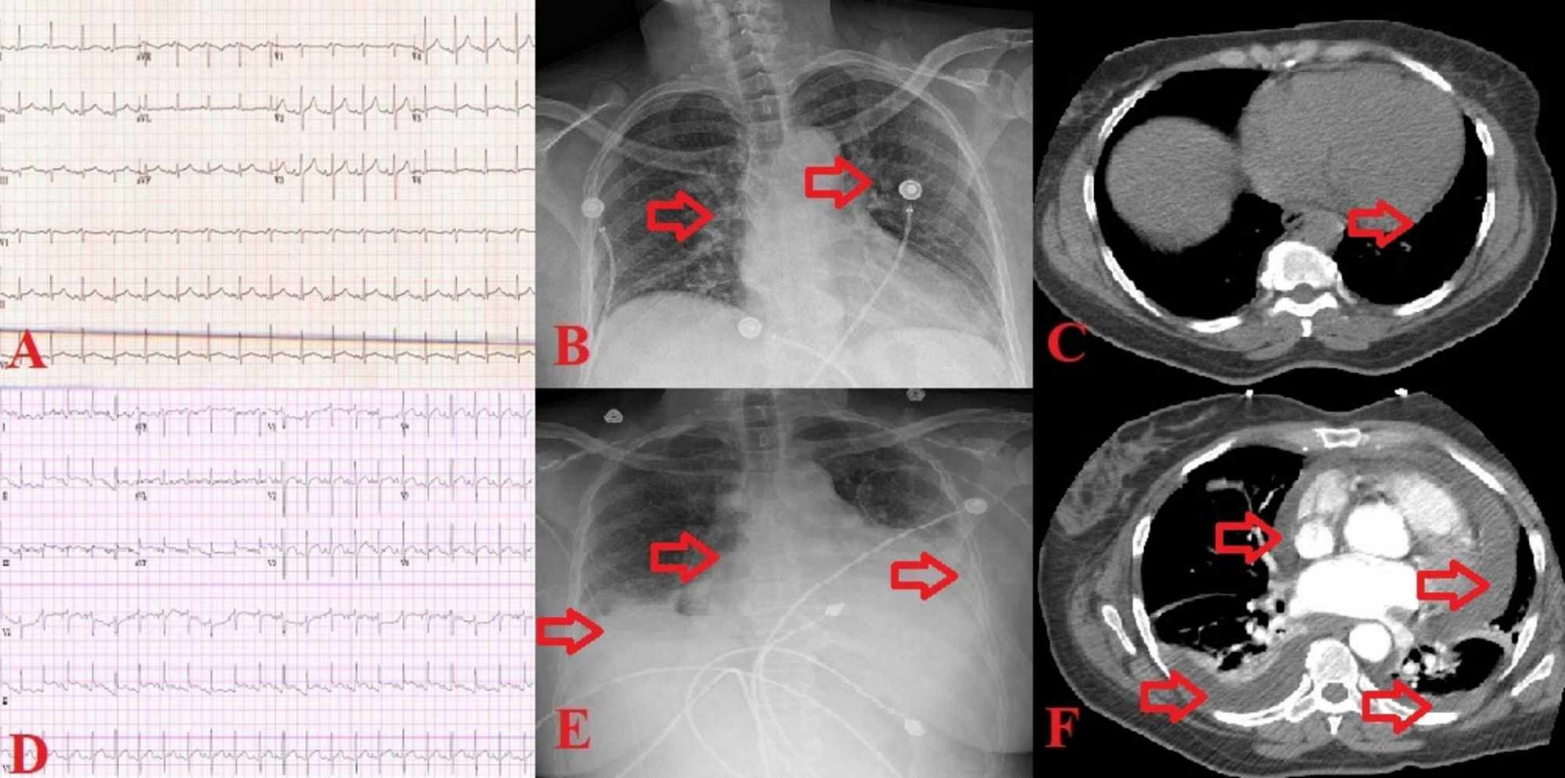
Images before developing the pericardial effusion and tamponade (top row, Panels A, B and C), and after (low row, Panels D, E and F). Panel A: EKG showing sinus tachycardia, 1 mm ST segment elevation in inferior leads. Panel B: Chest X-ray showing pulmonary venous congestion. Panel C: Chest CT showing mild pericardial effusion. Panel D: EKG showing sinus tachycardia, 1.5 mm ST segment elevation in inferior and lateral leads. Panel E: Chest X-ray showing left lower lobe consolidation, with greater left than right pleural effusion. Panel F: Chest CT showing expanding pericardial effusion, and mild pleural effusion.

**Figure 3:**
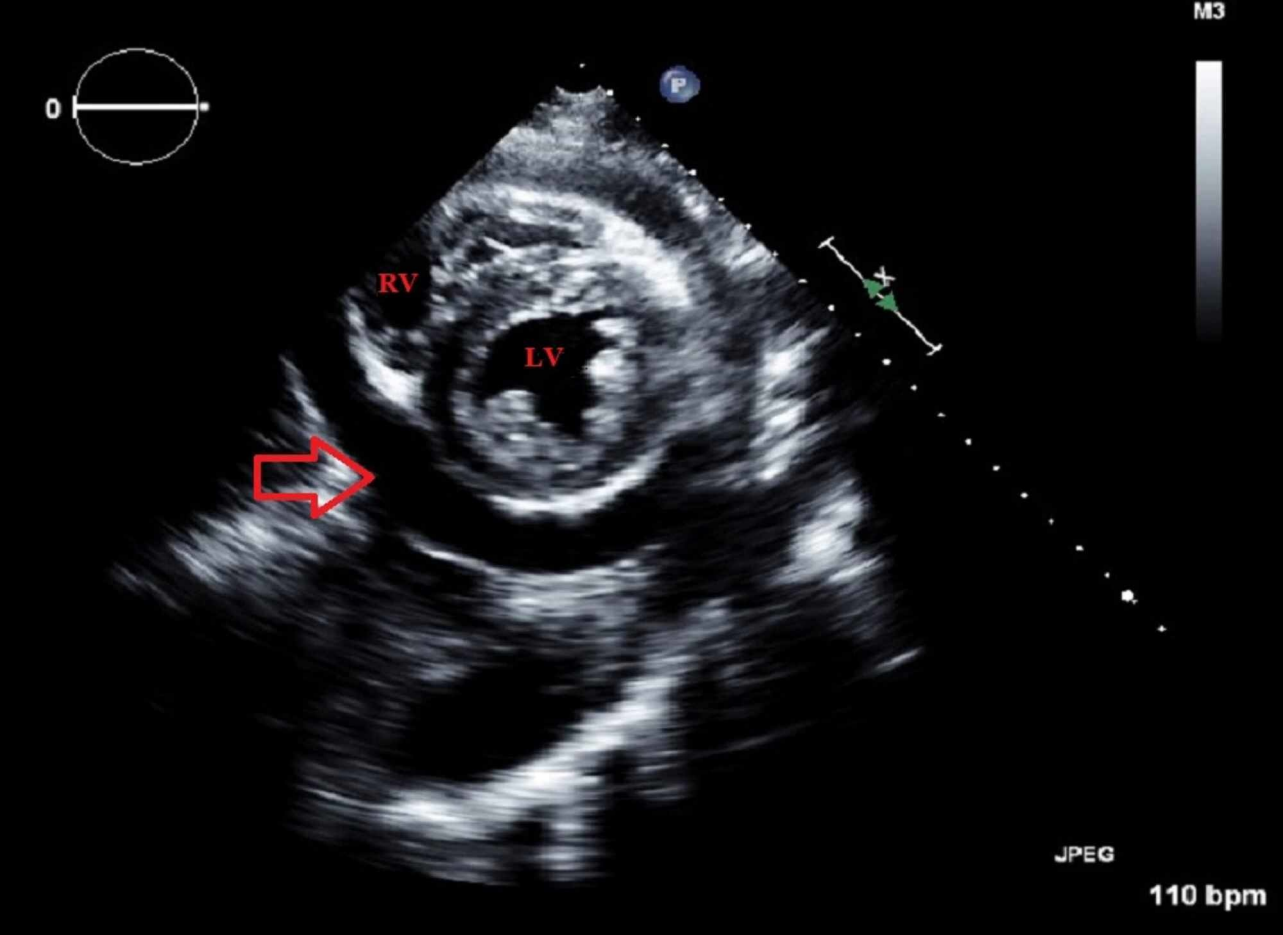
Short-axis TEE view, showing the left ventricle, right ventricle and a moderate-to-large pericardial effusion (arrow). TEE: Transthoracic echocardiogram

On the day of admission, electrocardiogram (EKG) (Figure 2, Panel A) showed sinus tachycardia, 1 mm ST segment elevation in inferior leads. Chest X-ray (Figure 2, Panel B) showed pulmonary venous congestion, but no cardiomegaly, no focal consolidations. Chest CT without intravenous contrast (Figure 2, Panel C) showed mild pericardial effusion, but was unremarkable otherwise. Four days later, she developed an acute hypoxic respiratory failure and had increased oxygen requirements reaching 8 L of oxygen-mask, and she was transferred to the medical intensive care unit (MICU). Exam showed tachycardia, tachypnea and increased JVD to 12-15 cm. EKG (four days after initial EKG, Figure 2, Panel D) showed sinus tachycardia, 1.5 mm ST segment elevation in inferior and lateral leads. Chest X-ray (four days after initial chest X-ray, Figure 2, Panel E) showed left lower lobe consolidation, with greater left than right pleural effusion. Transthoracic echocardiogram (TEE) was done and showed a moderate-to-large pericardial effusion. There were echocardiographic indications for tamponade. The inferior vena cava diameter was 2.1 cm with minimal respiratory variation. There was a circumferential pericardial effusion with a thickness of about 2 cm (Figure 3). Pericardiocentesis with a pericardial drain was then performed, fluid was yellow and cloudy, with WBC count of 302/mm^3^, 81% neutrophils, lactate dehydrogenase was 1339 unit/L. Pericardial fluid culture was positive for MRSA. Aspirin and colchicine were started.

Less than 48 hours after the drain was placed, it stopped draining and the patient had an increased JVD. At the time pulmonary embolism (PE) was to be ruled out. Thus, CT chest with intravenous contrast obtained (six days after initial CT, Figure 2, Panel F) showed an expanding pericardial effusion, and mild pleural effusion, but was negative for PE.

Attempts to exchange the drain failed. Cardiothoracic surgery felt she was not a surgical candidate, given her CD4 count. Condition was managed conservatively, thereafter, with six weeks of vancomycin, aspirin and colchicine.

## Discussion

MRSA pericarditis is a rare, potentially fatal disease. Route of infection is usually either by hematogenous or contiguous spread from retropharyngeal space, cardiac valves and below the diaphragm [11]. We believe route of infection in our case was by hematogenous spread, secondary to disseminated MRSA bacteremia due to untreated recurrent neck abscesses. Bacterial pericarditis typically presents with fever and chills, chest pain (often with dyspnea), and tachycardia. The presentation is always acute, with fevers occurring at regular intervals and frank rigors. Tachycardia is often due to the febrile response, but it may be an effort to compensate for decreased cardiac output from reduced ventricular filling due to cardiac tamponade. Tamponade can develop rapidly, as an effusion of 500 mL can accumulate over several days [12]. Chest pain is usually substernal pleuritic, often radiating to the scapular ridges due to the phrenic nerves passing through the anterior pericardium and innervating the trapezius ridges, which is consistent with our patient’s presentation [13]. Tuberculous pericarditis was also suspected given the patient’s severe immunosuppression, but acid-fast bacilli culture, and adenosine deaminase were negative which ruled out tuberculosis. Management of MRSA pericarditis was done through pericardial drain, with some cases treated with pericardial window (Class I recommendation for pericardial window, and Class IIa for subxiphoid pericardiotomy by 2015 ESC guidelines for the diagnosis and management of pericardial diseases: The task force for the diagnosis and management of pericardial diseases of the European Society of Cardiology) [14].

Our case was managed by pericardial drainage alone, and although she had recurrence of pericardial effusion, pericardiotomy was felt to be very risky given her AIDS with CD4 count <50. Thus, the team decided to proceed with medical management and pericardial drain insertion instead of the more aggressive option of pericardial window, and the patient’s condition improved with antibiotics, anti-inflammatory medications and pericardial drainage within days, in spite of recurrence of effusion and loculation, and the patient was discharged.

## Conclusions

MRSA pericarditis is a rare, possibly fatal, disease. It is usually a result of the spread of a remote infection (either hematogenously or contiguously). Prompt identification and diagnosis are key. Management is usually by drainage and/or pericardial window. Long-term intravenous antibiotics are usually required as well.
